# Supplementary material for: Anti-Phospholipid Antibodies in COVID-19 Are Different From Those Detectable in the Anti-Phospholipid Syndrome
Source: Front Immunol. 2020 Oct 15;11:584241. doi: 10.3389/fimmu.2020.584241 (PMC7593765; doi:10.3389/fimmu.2020.584241)
Supplement: Supplementary file 1 [file DataSheet_1.pdf]

Supplemental file reporting details on methods.

Anti-cardiolipin antibodies: aCL were detected by QUANTA Flash® Cardiolipin IgG/IgA/IgM (Inova Diagnostics, San Diego, CA) according to the manufacturer's instructions, as reported [16,17]. The cut-off values for anti-β2GPI were 20 chemiluminescent units (CU).

In-house ELISA was used for the detection of aCL IgG/IgM. Briefly, 96-well microplates, flat bottom (Nunc Immunoplate Polysorp) were coated with cardiolipin 50 μg/ml in absolute ethanol; 1:50 PBS-FBS 10% diluted sera were seeded in duplicate, and alkaline phosphatase (AP)-conjugated goat anti-human IgG/IgM antibody (Sigma) were used at 1:2000 - 1:5000 dilution in PBS-FBS 10%, respectively. Detection of antibody binding was carried out at 405 nm OD.

Anti-β2GPI antibodies: anti-β_2GPI antibodies were detected by QUANTA Flash® β_2GPI IgG/IgA/IgM (Inova Diagnostics) according to the manufacturer's instructions, as reported [16,17]. The cut-off values for anti-β2GPI were 20 chemiluminescent units (CU).

In-house ELISA was used for the detection of anti-β2GPI IgG/IgA/IgM. Briefly, human plasma β2GPI was purified by perchloric acid precipitation as described [16]. 96-well enhanced binding microplates, flat bottom (ThermoScientific) were coated with human purified β2GPI 10 μg/ml in calcium-carbonate buffer; 100 μl of 1:50 PBS-Tween20 0.05%$NaN_3$0.05% diluted sera were seeded in duplicate, and AP-conjugated goat anti-human IgG/IgA/IgM antibody (Sigma) were used at 1:2000 (IgG/IgA) or 1:5000 (IgM) dilution in PBS-Tween20 0.05%$NaN_3$0.05%. Detection of antibody binding was carried out at 405 nm OD.

Anti-D1 antibodies: anti-D1 IgG were tested by a chemiluminescent immunoassay exploiting the BIO-FLASH® technology (QUANTA Flash® β2GPI Domain 1 IgG; Inova Diagnostics) according to the manufacturer's instructions. The cut-off values for anti-D1 IgG positivity detected by QUANTA Flash were 20 chemiluminescent units (CU).

Anti-D4/5 antibodies: anti-D4/5 IgG were tested by in-house research ELISA assay. Briefly, 2.5 μg/ml of D4/5 peptides (kindly provided by Inova Diagnostics) in calcium-carbonate buffer were coated on flat bottomed enhanced binding microplates (ThermoScientific) at 4°C overnight. The plates were washed and blocked with PBS-BSA 1% $NaN_3$0.05% 1 hr at room temperature; 100 μl/well of the sera diluted 1:100 in PBS-Tween 0.05% $NaN_3$0.05% were added and incubated for 2 hrs at room temperature; after three further washes, 100 μl/well of AP-conjugated anti-human IgG (Sigma) diluted 1:800 in PBS/Tween 0.05% $NaN_3$0.05% were added and incubated for 1,5 hrs; after washing, the p-Nitrophenylphosphate substrate was added and antibody binding was evaluated at 405 nm. To assure inter-assay reproducibility, ELISA tests were carried out over a short time frame, in the same laboratory and under controlled conditions. Dilution curves of in-house positive standard and a low-positive reference sample were seeded for each run; the ELISA reaction was stopped at a fixed maximal OD values (1.40 OD). The inter-assay coefficient of variability was below 15%. Anti-D4/5 IgG results were expressed as optical density (OD) at 405 nm. Based on the previous analysis of 100 healthy individuals, the 95th percentile value was used to set the cut-off at 0.405 OD.
